# Comparison of the Effectiveness of Intravitreal Bevacizumab Injections with and without Simultaneous Cataract Surgery in Diabetic Patients with Macular Edema

**DOI:** 10.3390/jcm12124060

**Published:** 2023-06-15

**Authors:** Jeeyoung Kwak, You Hyun Lee, Kyung Tae Kang, Yu Cheol Kim

**Affiliations:** Department of Ophthalmology, Keimyung University School of Medicine, Daegu 42601, Republic of Korea; hyo01053@naver.com (J.K.); lyh8686@dsmc.or.kr (Y.H.L.); kkt0604@dsmc.or.kr (K.T.K.)

**Keywords:** bevacizumab, cataract extraction, diabetic retinopathy, macular edema, Irvine–Gass syndrome

## Abstract

Intravitreal bevacizumab (IVB), often injected during cataract surgery, is currently the main treatment for diabetic macular edema. This retrospective study aimed to compare the effectiveness of IVB injections alone and during cataract surgery in patients with diabetic macular edema. We examined 43 eyes in 40 patients who underwent cataract surgery with simultaneous IVB injections 3–12 months after IVB injections alone. Best-corrected visual acuity and central subfield macular thickness (CMT) were measured 1-month post-injection. The CMTs of the same eyes with IVB-only first and combined-treatment procedures later were 384 ± 149 vs. 315 ± 109 μm pretreatment (*p* = 0.0002), and after 1 month, they were 319 ± 102 vs. 419 ± 183 μm (*p* < 0.0001). In the IVB-only procedure, 56.1% of eyes had CMT < 300 μm 1 month after the injection compared to 32.5% after the combined treatment. Therefore, on average, when IVB was administered during cataract surgery, CMT increased, whereas after IVB injection alone, it effectively decreased. More prospective trials with large sample sizes are needed to evaluate the effectiveness of IVB injection performed simultaneously with cataract surgery.

## 1. Introduction

Diabetic macular edema (DME) results from the disruption of the blood–retina barrier (BRB) in diabetic patients [1]; it is conventionally defined as a retinal thickening or the presence of hard exudates within one disk diameter of the center of the macula [2]. DME is the most common cause of visual impairment among patients with diabetes, occurring in about 14% of this population [2,3]. BRB disruption leads to an accumulation of fluid and serum macromolecules in the intercellular space; in this pathological process, oxidative damage due to chronic hyperglycemia results in the overexpression of anti-vascular endothelial growth factor (VEGF) [2].

Thus, anti-VEGF therapy has become one of the main treatment options for DME in addition to laser photocoagulation treatment [2]. Bevacizumab is a drug widely used for this purpose, and intravitreal injection is occasionally performed simultaneously with cataract surgery for the patient’s convenience. Several studies have demonstrated that intravitreal bevacizumab (IVB) injection during cataract surgery prevents the progression of diabetic retinopathy more effectively than cataract surgery without IVB [4,5]. However, to the best of our knowledge, only a limited number of studies have compared the efficacy of IVB injection alone and when combined with cataract surgery. The purpose of this study was to analyze the effectiveness of IVB injection at the time of cataract surgery compared with the IVB-only injection.

## 2. Materials and Methods

### 2.1. Patients

This retrospective study adhered to the tenets of the Declaration of Helsinki and was approved by the Institutional Review Board of the Keimyung University Dongsan Hospital, Daegu, Korea (IRB no: 2022-09-014). We analyzed the medical records of patients who underwent cataract surgery with simultaneous IVB injections between August 2017 and January 2022 at the Keimyung University Dongsan Hospital. The requirement for informed consent was waived by the IRB due to the retrospective nature of the study.

The inclusion criteria were clinically significant macular edema and diabetic retinopathy (DR) in phakic eyes and history of IVB-only injection 3–12 months before IVB during cataract surgery. Thus, we analyzed the same group of eyes treated at first with IVB only and subsequently with IVB and cataract surgery, comparing the effects of the different procedures.

The exclusion criteria were advanced proliferative DR, massive vitreous hemorrhage, tractional membranes, any other retinal diseases (such as branch retinal vein occlusion, ocular ischemic syndrome, and age-related macular degeneration), or a history of ocular trauma or surgery.

### 2.2. Surgical Procedure

All bevacizumab doses for intravitreal injections were prepared in approximately 20 individual syringes from each 4 mL vial by the Chemotherapy Department of the hospital pharmacy, as recommended by the American Academy of Ophthalmology Risk Management Guidelines, to avoid vial contamination with each dose extraction. Each syringe contained 0.15–0.18 mL of bevacizumab solution for a 0.05 mL injection (1.25 mg of bevacizumab), and the syringes were delivered aseptically to the Ophthalmology Clinic and the operating room.

All cataract surgeries were performed by two surgeons (K.Y.C. and K.K.T.) and included the process of phacoemulsification, with a Centurion^®^ Vision System (Alcon, Geneva, Switzerland), and the implantation of an acrylic intraocular lens (Vivinex iSert^®^, Hoya Surgical Optics, Tokyo, Japan) in the capsular bag under topical anesthesia. In the combined procedure, intravitreal injection of 1.25 mg of bevacizumab was performed via the pars plana using a 30-gauge needle in the superotemporal area after intraocular lens insertion and before the ocular viscoelastic devices (OVDs) were removed by irrigation and aspiration (I&A). All patients were prescribed moxifloxacin and prednisolone acetate 1% eye drops postoperatively every 2 h for 1 week, tapered to 4 times a day for the following 3 weeks. 

In contrast, the IVB-only injection was administered at the Ophthalmology Clinic after the doctor’s examination. Similarly to the combined procedure, 1.25 mg of bevacizumab was injected intravitreally via the pars plana using a 30-gauge needle in the superotemporal area aseptically. The patients were prescribed moxifloxacin eye drops every 2 h for 1 week after the injection.

### 2.3. Pre- and Postoperative Examinations

All patients underwent best-corrected visual acuity (BCVA) measurements and slit-lamp examinations at each visit. The retina was assessed using a 90-diopter lens and fundus photography (Optos Ultra-widefield^®^, Optos, Dunfermline, UK). The central subfield macular thickness (CMT) was measured within a 1 mm diameter circle centered on the fovea using swept-source optical coherence tomography (SS-OCT) (DRI-OCT Triton^®^, Topcon, Tokyo, Japan). BCVA and CMT were measured before and 1 month after the injections. The effects on the CMT of the IVB-only injection (IVB-only group) and IVB injections during cataract surgery (combined-procedure group) were compared and analyzed.

### 2.4. Statistical Analysis

All statistical analyses were performed using IBM SPSS software (version 18.0; IBM, Armonk, NY, USA). The normal distribution of each parameter in the two procedure groups was confirmed using Kolmogorov–Smirnov and Shapiro–Wilk tests. A paired *t*-test was used to compare BCVA and CMT pre- and post-injection in both IVB-only and combined-procedure groups. The chi-square test was used to compare the proportion of eyes with CMT < 300 μm and increased CMT after each procedure. Statistical significance was set at *p* < 0.05. The statistical power of the mean difference in CMT between the IVB-only and combined-procedure groups was 85.9%, and other significant cases were distributed from 63.0% to 77.3%.

## 3. Results

In total, 43 eyes from 40 patients with DME were included in this study, among 179 eyes that received IVB at the time of cataract surgery from August 2017 to January 2022 in our hospital. However, only 43 eyes met the inclusion criteria. In this study, 48 eyes with advanced proliferative diabetic retinopathy requiring vitrectomy were excluded; 47 eyes did not have a previous IVB injection, and 24 eyes had a previous injection outside of the time interval considered (3–12 months before the combined procedure). Twelve eyes lacked OCT data, and five eyes were from patients with other diseases (age-related macular degeneration or branch retinal vein occlusion).

The mean age of the patients was 62.5 ± 10.5 years. Patient demographics are summarized in Table 1. All eyes had DR at baseline, ranging from mild non-proliferative to proliferative (Table 2). A vitreous hemorrhage occurred in one eye after IVB injection combined with cataract extraction; in this case, the BCVA was 10/50 in Snellen at postoperative day (POD) 1, 20/50 at POD 3, 30/50 at POD 10, and reached 20/20 at POD 20. The intraocular pressure was 16 mmHg at POD 1, 14 mmHg at POD 3, 17 mmHg at POD 10, and 14 mmHg at POD 20. No other complications occurred after surgery or injection. The mean BCVA (logMAR, Snellen) and CMT measured with OCT were recorded before and 1 month after each injection (IVB-only and combined procedure).

In the IVB-only group, the mean BCVA was 0.541 ± 0.361 logMAR (0.371 ± 0.235 Snellen) prior to treatment and 0.540 ± 0.305 logMAR (0.352 ± 0.236 Snellen) after 1 month (*p* < 0.001 using both scales). The mean baseline CMT was 384 ± 149 μm at baseline and 319 ± 102 μm (*p* < 0.001) after 1 month. Therefore, BCVA improved slightly, whereas CMT significantly decreased after the injection.

**Figure 1:**
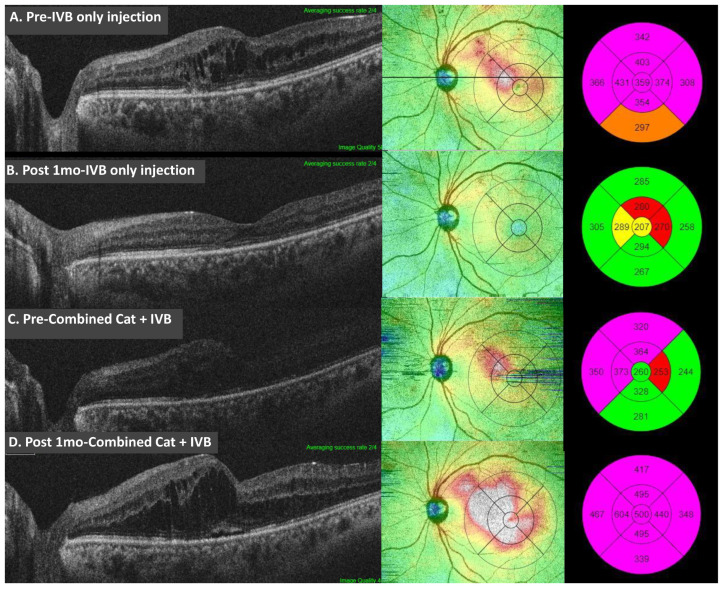
OCT images of a representative patient with diabetic maculopathy before and 1 month after intravitreal bevacizumab (IVB)-only injection and IVB injection combined with cataract surgery. The mean central macular thickness showed a significant decrease from (**A**) 359 μm to (**B**) 207 μm after the IVB-only injection and a significant increase from (**C**) 260 μm to (**D**) 500 μm after the IVB injection combined with cataract surgery in the same patient.

In contrast, for the combined-procedure group, the mean BCVA at baseline was 0.631 ± 0.339 logMAR (0.293 ± 191 Snellen) and 0.368 ± 0.265 logMAR (0.492 ± 0.245 Snellen) after 1 month (*p* = 0.01). The mean CMT was 315 ± 109 μm at baseline, and it increased to 419 ± 183 μm (*p* < 0.0001) 1-month post-injection. BCVA significantly improved after the combined procedure, whereas CMT increased significantly. Figure 1 shows the OCT feature with a significant decrease in CMT after the IVB injection alone and the increased CMT instead after the IVB injection combined with cataract surgery. The results are summarized in Table 3.

Table 4 and Table 5 summarize the proportion of eyes with CMT < 300 μm and increased CMT after each injection, respectively. Due to a lack of OCT data, two eyes were excluded from the IVB-only group, and three eyes were excluded from the combined-procedure group. Additionally, one eye was excluded due to vitreous hemorrhage after the combined procedure preventing OCT acquisition. The number of eyes with CMT < 300 μm was 12/41 (29.3%) at baseline and 23/41 (56.1%) after the injection in the IVB-only group (*p* = 0.003). In the combined procedure, the numbers were 23/40 (57.5%) before the procedure and 13/40 (32.5%) afterward (*p* = 0.002). The number of eyes with increased CMT after the injection, instead of decreased, was 11/41 (26.83%) in the IVB-only group (*p* = 0.031) and 33/41 (82.5%) in the combined-procedure group (*p* = 0.432). In a subgroup analysis, comparing the eyes with decreased versus increased CMT after the combined procedure, the mean intervals between the two injections were 5.02 ± 0.58 and 5.57 ± 0.77 months and not significantly different (*p* = 0.275). Surgical factors, such as cumulative dissipated energy (CDE) and surgical time, were also compared; the surgical time was defined as the time from the first incision to the removal of the speculum. In the decreased versus increased CMT subgroups, the CDE was 7.13 ± 2.22 vs. 8.40 ± 6.90%s, showing no significant difference (*p* = 0.126). The surgical time was 15.0 ± 3.46 vs. 16.84 ± 6.77 min and also not significantly different.

Among the 33 eyes with increased CMT after the combined procedure, 16 had additional intravitreal (IV) injections within 2 months. Fourteen eyes were treated with additional IV triamcinolone acetonide (TA): one with IVB and one with IV aflibercept (IVA). The eyes that received additional IVTA after the surgery showed an average CMT decrease of 263.29 ± 149.65 μm. The two eyes that were treated with additional IVB and IVA after surgery showed a CMT decrease of 127 and 184 μm, respectively.

## 4. Discussion

Our results revealed that the CMT generally decreased when IVB was injected alone, whereas it increased when the injection was administered during cataract surgery. Additionally, the proportion of eyes with CMT < 300 μm was larger after the IVB-only injection than after the combined procedure. The baseline mean CMT before the two procedures was significantly different (IVB-only injection: 384 μm; combined procedure: 315 μm); however, the results were inverted after the injections (IVB-only injection: 319 μm; combined procedure: 419 μm). Thus, the effect of the IVB injection in reducing CMT was superior when administered alone. Furthermore, we investigated the proportion of eyes with increased CMT after the IVB-only injection and combined procedure. The latter group had a higher percentage of eyes with increased CMT, rather than decreased CMT, after the injection compared to the IVB-only group.

Several studies have shown that cataract surgery increases the risk of DME due to alterations in the ocular microenvironment and elevated inflammatory cytokines, causing an imbalance between angiogenic factors [6]. The reported rate of DR progression after cataract surgery ranges from 20% to 45% [5,7] and varies based on diabetes control status, renal function, hypertension, postoperative inflammation degree, and DR severity. The intraoperative IVB treatment during cataract surgery in DR is known to be effective in preventing DR progression in the short term, reducing the rate to 7.4–11.4% [4,5,7].

However, our results show that the IVB injection administered alone is more effective than when combined with cataract surgery. BCVA and CMT were evaluated before the injection and 1 month afterward because the half-life of the drug is 4 weeks, as reported in a retinal penetration study [8]. The indication for cataract surgery in this study was a significant visual disturbance caused by lens opacity, which is associated with a stable macular thickness after IVB injection.

A possible reason for the reduced effect of IVB when administered during cataract surgery might be the surgery itself. The optimal timing for the IVB injection within the surgery has yet to be established; in our study, the injection was performed after intraocular lens insertion and before OVD removal with I&A. It is possible that some of the drugs were removed with the OVDs during I&A. Performing the injection at the end of the surgery may be a more suitable option; however, further studies are needed to confirm this hypothesis.

Another possible reason is the aggravation of macular edema after the surgery. The possible occurrence of Irvine–Gass syndrome should be considered since distinguishing between DME and pseudophakic cystoid macular edema (CME) can be difficult. Trauma related to surgery acts as a proinflammatory stimulation, altering the ocular microenvironment and increasing the level of cytokines, such as VEGF, interleukin-1b (IL-1b), IL-6, and monocyte chemotactic protein-1 [6,9]. In addition, prolonged postoperative inflammation leads to ruptures in the BRB and consequently increases oxidative stress [9]. Moreover, when the vitreous structure is deformed after cataract surgery, its traction force is transmitted to the macula or the optic nerve, causing CME [9]. It is still unclear whether the DR progression after cataract surgery is due to its natural course or the effect of the surgery itself [10,11]; the observation period was relatively short in this study (6 months to 1 year), and the proinflammatory effect of the surgery seemed to increase DME [9].

On the other hand, the improvement in BCVA was greater with the combined procedure than with IVB injection alone. This effect is likely due to the treatment of lens opacity, despite the greater macular edema reduction with the IVB-only injection. However, possible complications should be considered when IVB is injected during cataract surgery. One of the forty-three eyes in our study incurred vitreous hemorrhage after surgery. When the needle was inserted, the eye was slightly distorted due to transient hypotony with an unstable anterior chamber state. The hemorrhage spontaneously resolved in 2 weeks. 

Among the 33 eyes with increased CMT after the combined procedure, 16 were treated with additional injections. Most eyes were treated with IVTA when no decrease in CMT was noticed after the combined procedure; this treatment was chosen because of its cost-effectiveness, history of failure with IVB, and no risk of cataract formation. 

In this study, the parameters related to the two types of injection were compared at different times in the same group of patients. Therefore, there was no bias due to differences in patient characteristics, such as diabetes duration and control status, hypertension, or renal dysfunction. In addition, the interval between the two injections was limited to 3–12 months, and all patients underwent cataract extraction in stabilized DR conditions.

This study had some limitations. First, it was retrospective and had a small sample size. Second, as mentioned above, the eyes before the two types of IVB injections had significantly different CMT and BCVA, which is a limitation due to passing time. Third, IVB was injected before I&A during the cataract surgery, possibly causing some loss of bevacizumab while removing the remnant OVDs. The drug effect might be greater if the injection is performed at the end of surgery; further studies addressing this limitation are needed.

In conclusion, IVB appears to be less effective when injected during cataract extraction (before I&A) than alone. In addition, when IVB injection is administered during surgery, bleeding may occur at the injection site due to a relatively low eye tone, possibly causing sight-threatening side effects, such as vitreous hemorrhage or ciliary body dehiscence.

## Figures and Tables

**Table 1:** Baseline characteristics of the patients.

	Patients (*n* = 40, 43 Eyes)
Age (years)	62.5 ± 10.5
Sex (M, F)	28, 15
Eye (right, left)	22, 21
DM duration (years)	13.1 ± 7.71
Medication (PO, insulin)	31, 12
Hypertension	14 (32.56%)
CKD	10 (23.26%)
Hemodialysis	8 (18.6%)
Lens grade	
NO, NC 2	2 (4.65%)
NO, NC 2.5	5 (11.63%)
NO, NC 3	29 (67.44%)
NO, NC 3.5	2 (4.65%)
NO, NC 4	4 (9.30%)
NO, NC 5	1 (2.33%)
Injections interval (months)	5.21 ± 0.47

The data are expressed as numbers, number and percentage, or mean ± standard deviation as appropriate. DM: Diabetes mellitus; PO: per os; CKD: chronic kidney disease; NO: nuclear opalescence; NC: nuclear color.

**Table 2:** Grade of diabetic retinopathy and mean central macular thickness after the IVB-only injection and combined procedure.

	IVB	Cat + IVB
Retinopathy		
Mild NPDR	1 (2.33%)	
Mod NPDR	2 (4.65%)	
Severe NPDR	7 (16.28%)	
PDR	33 (76.74%)	
Maculopathy		
CSME	43 eyes (100%)	
Mean CMT before injection (μm)	384 ± 149	315 ± 109
Free period * (months)	3.74 ± 0.44	3.55 ± 0.51

* The free period was defined as the interval before another injection when no treatment was required. The data are expressed as numbers and percentages or mean ± standard deviation as appropriate. IVB: Intravitreal bevacizumab; Cat: cataract surgery; NPDR: non-proliferative diabetic retinopathy; PDR: proliferative diabetic retinopathy; CSME: clinically significant macular edema; CMT: central macular thickness.

**Table 3:** Central macular thickness and best corrected visual acuity before and 1 month after injections.

	IVB only	Cat + IVB	*p* *
CMT			
Before	384 ± 149	315 ± 109	0.0002
After 1 month	319 ± 102	419 ± 183	<0.0001
*p*	<0.001	<0.001	
BCVA logMAR			
Before	0.541 ± 0.361	0.631 ± 0.339	0.084
After 1 month	0.540 ± 0.305	0.368 ± 0.265	<0.001
*p*	<0.001	0.01	
BCVA Snellen			
Before	0.371 ± 0.235	0.293 ± 0.191	0.011
After 1 month	0.352 ± 0.236	0.492 ± 0.245	<0.001
*p*	<0.001	<0.001	

The data are expressed as mean ± standard deviation. IVB: Intravitreal bevacizumab; Cat: cataract surgery; CMT: central macular thickness; BCVA: best corrected visual acuity. *: the paired *t*-test was used to analyze the differences between the two procedures.

**Table 4:** Proportions of eyes with CMT < and > 300 μm after the IVB-only and combined procedure.

CMT	<300 μm	>300 μm	*n*	*p* *
Pre-IVB only Post-IVB only	12 (29.3%) 23 (56.1%)	29 (70.7%) 18 (43.9%)	41 (100%) 41 (100%)	0.003
Pre-combined procedure Post-combined procedure	23 (57.5%) 13 (32.5%)	17 (42.5%) 27 (67.5%)	40 (100%) 40 (100%)	0.002

CMT: Central macular thickness; IVB: intravitreal bevacizumab; * *p*: chi-square test.

**Table 5:** Proportions of eyes with increased CMT after the IVB-only and combined procedure.

CMT	Pre < 300 μm	Pre > 300 μm	*n*	*p* *
IVB-only Decreased CMT (Pre > Post) Increased CMT (Pre < Post)	12 6 (50%) 6 (50%)	29 24 (82.76%) 5 (17.24%)	41 30 (73.17%) 11 (26.83%)	0.031
Combined procedure Decreased CMT (Pre > Post) Increased CMT (Pre < Post)	23 3 (13.04%) 20 (86.96%)	17 4 (23.53%) 13 (76.47%)	40 7 (17.5%) 33 (82.5%)	0.432

CMT: Central macular thickness; IVB: intravitreal bevacizumab; * *p*: chi-square test.

## Data Availability

The data that support the findings of this study are available upon request from the corresponding author. The data are not publicly available due to privacy or ethical restrictions.
